# Hereditary Hemorrhagic Telangiectasia in a Young Adult: Gastrointestinal Arteriovenous Malformations as a Presenting Feature

**DOI:** 10.7759/cureus.43915

**Published:** 2023-08-22

**Authors:** Jayasree Ravilla, Ping He, Anish Patel

**Affiliations:** 1 Internal Medicine, Monmouth Medical Center, New Jersey, USA; 2 Gastroenterology, Rutgers Robert Wood Johnson Medical School, New Brunswick, USA

**Keywords:** mutations, gastrointestinal bleeding, avm, hht, epistaxis

## Abstract

Hereditary hemorrhagic telangiectasia (HHT) is characterized by the presence of multiple arteriovenous malformations (AVM) in which arteries and venules are connected directly without intervening capillaries. The primary clinical presentation is spontaneous and recurrent nosebleeds (epistaxis), typically starting around 12. Telangiectases, which are small arteriovenous malformations, are commonly found in mucocutaneous areas and gastrointestinal (GI) mucosa. The presentation of epistaxis precedes the occurrence of telangiectases. Larger AVMs most commonly affect the lungs, liver, or brain, and complications from bleeding or shunting can be potentially life-threatening. Here, we discuss the case of a 44-year-old man who presented to the emergency department with a chief complaint of fatigue for three weeks associated with shortness of breath. His eventual labs revealed severe anemia with a hemoglobin of 4.4 g/dL. He denied any history of symptoms of GI bleeding. Computed tomography of the abdomen was done which did not reveal any intra-abdominal bleeding, hematoma, or abnormality. Further history revealed a recent diagnosis of HHT in his mother through genetic testing. During the stay, he also developed spontaneous epistaxis which was treated with topical vasoconstrictors by the ear, nose, and throat (ENT) physician. Given the high likelihood of HHT, he underwent luminal evaluation. Upper and lower endoscopies of the gut revealed multiple AVMs. A diagnosis of HHT was established as he fulfilled the criteria required for the diagnosis of HHT. It is rare for individuals with HHT to experience GI bleeding before the age of 50, making this case unique.

## Introduction

Gastrointestinal arteriovenous malformations (AVMs) comprise less than 5% of all the sources of gastrointestinal (GI) bleeding. Although the etiology of AVMs remains unclear, they are found to be frequently observed in conditions such as hereditary hemorrhagic telangiectasia (HHT), Phosphatase and Tensin Homolog deleted on Chromosome 10 (PTEN) syndrome, and genetic abnormalities like Ras p21 protein activator 1 (RASA1) mutations [1]. HHT, or Osler-Weber-Rendu disease, is an autosomal dominant disorder resulting from a mutation in certain genes resulting in vascular malformations in various mucocutaneous and visceral organs. Individuals with HHT are found to have mutations in proteins like endoglin (ENG) in HHT1, activin receptor-like kinase 1 (ACVRL1/ALK1) in HHT2, and mothers against decapentaplegic homolog 4 (SMAD4) which is linked to HHT in conjunction with juvenile polyposis (JP) along with abnormal levels of transforming growth factor-beta (TGF-β) and vascular endothelial growth factor (VEGF) in the plasma. The majority of individuals diagnosed with HHT have a confirmed affected parent upon thorough medical history. The Curaçao criteria are commonly used for diagnosis and include family history, recurrent epistaxis, cutaneous telangiectasia, and the development of internal organ arteriovenous malformations [2]. GI telangiectasias are commonly observed in a significant proportion of patients (around 70%), and they can potentially result in hemorrhages and anemia [3,4]. The stomach and small intestine are the common locations for GI telangiectases in patients with HHT. The management approach involves distinguishing between different organ locations and managing them symptomatically based on their specific locations. Generally, symptomatic telangiectases of the skin, oral and GI mucosa, and liver are treated based on symptom severity but, in the case of AVMs in the lungs and brain, treatment is recommended even in the absence of symptoms due to their potential for sudden and catastrophic presentation [5,6].

## Case presentation

A 44-year-old male with no past medical history presented to the emergency department with a three-week duration of fatigue associated with severe shortness of breath with exertion. He never had similar complaints in the past. He denied any allergies and use of illicit substances. On admission, he was hypotensive with blood pressure (BP) of 88/62 mm Hg which improved after intravenous (IV) fluid boluses. The physical exam showed no abdominal tenderness, and the skin examination was without evidence of a rash, ecchymosis, or telangiectasias. Blood work revealed hemoglobin (Hb) of 4.4 g/dL with a hematocrit of 28%, blood urea nitrogen (BUN) of 16 mg/dL, and creatinine of 0.9 mg/dL (Table 1). He was given four blood transfusions which improved his Hb to 8.2 g/dL. He underwent computed tomography (CT) of the chest, abdomen, and pelvis to look for the cause of anemia. His CT scan did not reveal any obvious pathology or signs of bleeding, no AVMs were noted in the chest and abdomen.

**Table 1:** Laboratory test results on the day of admission

Laboratory test result	Value
White blood cell (4,000-11,000 cells/mm^3^)	10000 cells/mm^3^
Red blood cells (4.7-6.1 million cells/mcL)	4.2 million cells/mcL
Hemoglobin (11-15.1 g/dL)	4.4 g/dL
Hematocrit (33.1%-44.5%)	29%
Platelet (150,000-400,000 cells/mm^3^)	186000 cells/mm^3^
Mean corpuscular volume (80-100 fL)	78 fL
Red cell distribution width (12%-15%)	16.8%
Serum iron level (45-135 ug/dL in men)	21 ug/dL
Transferrin saturation (20%-50%)	6%
Total iron binding capacity (250-400 ug/dL)	332 ugdL
Ferritin (24-336 ng/mL)	10.4 ng/mL
Blood urea nitrogen (6-20 mg/dL)	15 mg/dL
Creatinine (0.44-1.03 mg/dL)	0.8 mg/dL
Total bilirubin (0.3-1 mg/dL)	0.7 mg/dL
Alkaline phosphatase (32-91 U/L)	89 U/L
Aspartate aminotransferase (15-41 U/L)	22 U/L
Alanine aminotransferase (7-52 U/L)	17 U/L
Lactic acid (< 2 mmol/L)	1.2 mmol/L
International normalized ratio (0.8-1.1)	1.66

**Figure 1:**
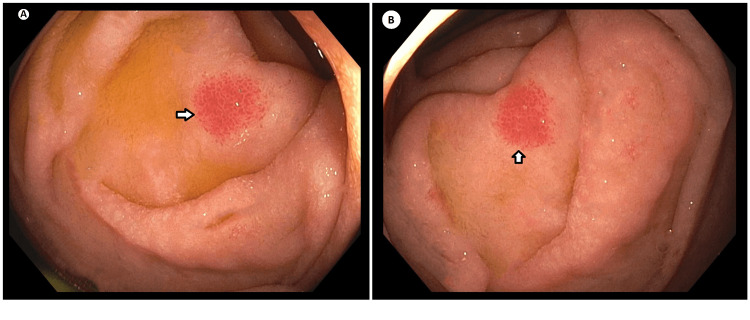
EGD showing the second part of the duodenum (A & B) Non-bleeding angioectasia in the duodenum (arrows in A & B) EGD: esophagoduodenoscopy

**Figure 2:**
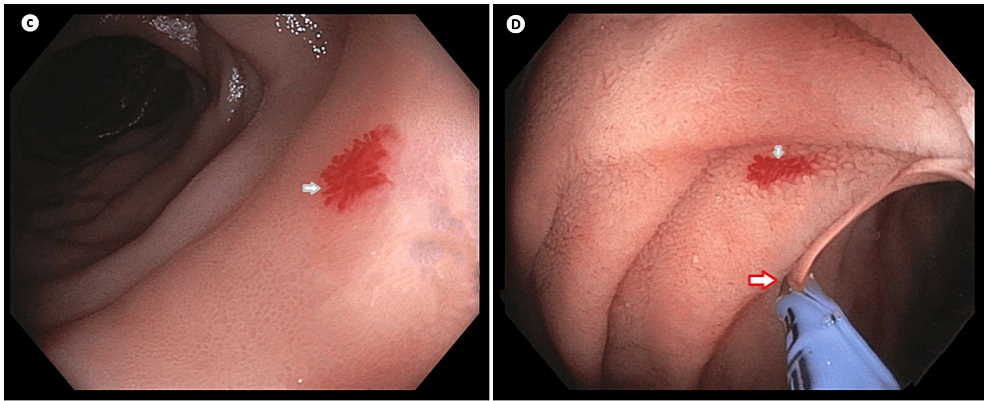
Push enteroscopy showing jejunum part Non-bleeding AVMs of jejunum in both C & D (represented by grey arrows) Hemostatic clipping applied to angioectasia of jejunum in image D (represented by red arrow) AVM: arteriovenous malformation

During his third day of hospitalization, he experienced spontaneous nosebleeds (epistaxis) which necessitated a nasal endoscopy and the application of a topical vasoconstrictor. While in the hospital, he was evaluated by hematology to rule out any bleeding disorders (Table 2) and the initial serologic workup was unrevealing. Further investigation revealed a recent diagnosis of HHT in the mother through genetic testing and she had a history of epistaxis. He then underwent luminal evaluation by gastroenterology to determine the etiology of low Hb. Esophagoduodenoscopy (EGD) with push enteroscopy revealed numerous AVMs in the small bowel (duodenum and jejunum as depicted in Figures 1-2). These were treated with the application of argon plasma coagulation (APC).

**Table 2:** Labs for bleeding disorders

Laboratory test result	Result
PROTEIN C (70-150%)	normal
PROTEIN S (60-150%)	normal
Factor V (50%-200%)	normal
Factor VII (65-180%)	normal
Factor X (45-1505%)	normal
Factor VIII (50-150%)	normal
Factor IX (50-150%)	normal
Von willebrand factor (50-200 IU/dL)	normal
Vitamin K level (0.2-3.2 ng/mL)	normal
Prothrombin time (11-13.5 sec)	13 sec
Partial thromboplastin time (PTT - 25-35 sec)	27.5 sec
Bleeding time (2-9 minutes)	6 minutes
d dimer (< 500 ng/ml)	2035 ng/mL
Fibrinogen (200-400 mg/ dL)	309 mg/dL

**Figure 3:**
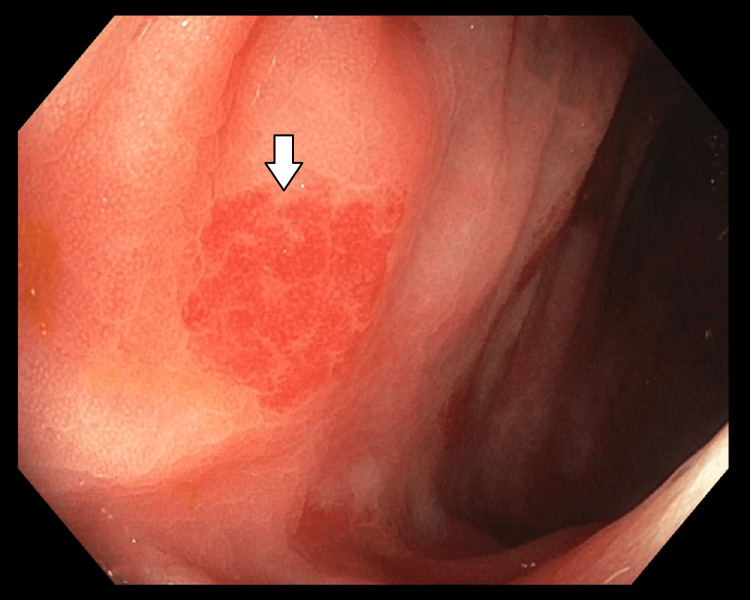
Colonoscopy showing the ascending part of the colon Non-bleeding AVM along the ascending colon (represented by white arrow) AVM: arteriovenous malformation

**Figure 4:**
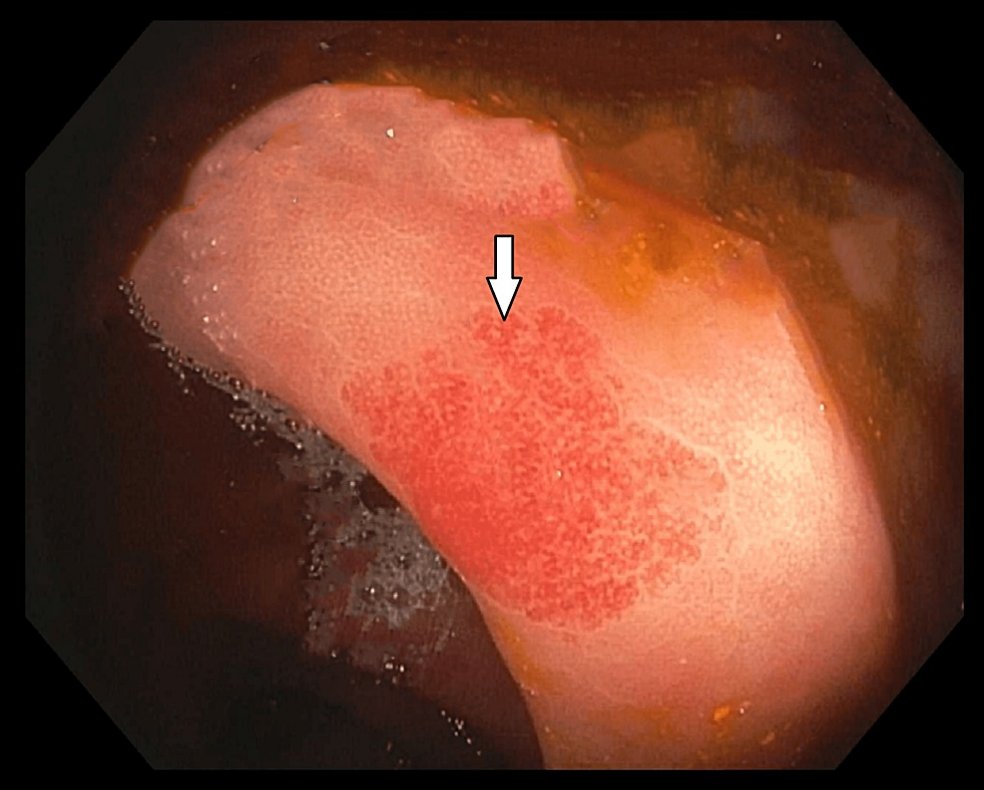
Colonoscopy showing the sigmoid colon Another non-bleeding AVM in the sigmoid colon (white arrow) AVM: arteriovenous malformation

Subsequent colonoscopy revealed multiple AVMs all throughout the colon (Figures 3-4). These were also treated with the application of APC. No upper GI or colonic malignancy was identified. As patient met the clinical criteria for HHT: spontaneous epistaxis, multiple visceral telangiectasias with GI involvement, and a first-degree relative with a confirmed diagnosis of HHT. Given he met three out of four Curaçao diagnostic criteria for HHT, he was diagnosed with definitive HHT. He was discharged with outpatient GI follow-up. He was instructed for genetic testing in the future.

## Discussion

HHT, an autosomal disorder, exhibits a diverse range of clinical manifestations [7]. Around one to two-thirds of patients require medical treatment [8]. The symptoms originate from a generalized structural abnormality in the small blood vessels, leading to the formation of arteriovenous anastomoses which manifest as telangiectatic lesions in the skin and mucosa [7]. The most common symptoms of HHT are nosebleeds (epistaxis), GI bleeding, and anemia caused by telangiectasias affecting the skin and mucous membranes. However, HHT patients also face the risk of developing AVMs in the cerebral, pulmonary, and hepatic circulations, which can result in damage to vital organs. The initial screening method for HHT is the Curaçao criteria as mentioned earlier and treatment primarily involves managing symptoms and complications, as well as early screening for AVMs [7-9].

Almost 96% of patients with HHT exhibit genetic mutations in either the *ENG* or *ACVRL1* genes, which are a part of the TGF-β pathway. Patients with both JP and HHT (JP-HHT) exhibit mutations in *SMAD4*, another member of the TGF-β pathway. Two other new genes *SMAD4* and growth differentiation factor (GDF2) are identified in fewer patients exhibiting a similar or overlapping phenotype to HHT. The major cause for these disorders seems to be the mutations in multiple genes involved in the TGF-β signaling pathway that have an active role in various cellular processes such as proliferation, differentiation, apoptosis, and migration. The genes responsible for specific HHT subtypes include *ENG* (located on chromosome 9q34) for HHT1, *ACVRL1/ALK1* (located on chromosome 12q13) for HHT2, and *SMAD4* (located on chromosome 18q21) for the combined JP/HHT syndrome [10,11].

Epistaxis is the earliest and most prevalent symptom of HHT beginning around the age of 11. Epistaxis occurred in 91% of HHT1 cases compared with 96% of HHT2 cases. Visceral lesions are usually diagnosed in adults with median ages of pulmonary AVMs (PAVM) and GI AVMs being around 35.7 and 55.4 years respectively. PAMs, which present as dyspnea, cyanosis, and clubbing, were commonly associated with HHT1 (61%) as compared to HHT2 (17%). Complications of PAVMs include pulmonary hypertension (HTN) and hemothorax. Cerebral AVMs (CAVM) were found only in people with HHT1 and can present as stroke and transient ischemic attack (TIA). Hepatic AVMs (HAVM) are more significant in HHT2 (83%) than HHT1 (60%) with presenting features of portal HTN, cirrhosis, ascites, and right-sided heart failure. GI involvement was not significantly different between HHT1 and HHT2. The most common complications of HHT are bleeding and anemia with epistaxis and GI bleeding being the common frequent findings although bleeding can be rarely observed in the liver and urinary tract. In young patients with HHT and pulmonary AVM, strokes are primarily caused by paradoxical embolism [12]. In GI involvement, colonic polyps were found in 97% of the adults who underwent colonoscopy. The occurrence of gastric and small-bowel polyps was lower compared to colonic polyps and SMAD4 mutations are more likely to be associated with extensive gastric polyposis than bone morphogenetic protein receptor (BMPR1A) mutations [13,14].

The assessment of PAVMs is done using transthoracic contrast echocardiography (TTCE) and CT examinations in patients with HHT. Contrast-enhanced magnetic resonance angiography (MRA) is currently not used for PAVMs screening but can be a valuable tool for pre-embolization planning [15]. Cerebral angiography, including super-selective angiography, is definitive for diagnosing all CAVMs, including micro AVMs, in patients with HHT. MR images may underestimate the occurrence of cerebral vascular malformations in HHT patients [16]. Around 25-33% of individuals with HHT experience GI bleeding at the location of telangiectases, leading to morbidity from severe anemia and a need for frequent blood transfusions. Telangiectases are usually numerous and present throughout the GI tract [17]. For GI AVMs, colonoscopy, EGD, video capsule endoscopy, abdominal Doppler ultrasound of the liver, and subsequent CT scan or MRI based on the specific manifestations are the mainstay of diagnosis [3]. The diagnosis of HHT is determined by Curaçao criteria (Table 3) [3].

**Table 3:** Curaçao criteria HHT: hereditary hemorrhagic telangiectasia

S. No	Criteria
1	Recurrent and spontaneous nosebleeds
2.	Presence of telangiectasias at characteristic locations
3	The existence of visceral arteriovenous malformations or telangiectasias
4	family history of HHT in a first-degree relative, which is typically inherited in an autosomal dominant manner

Patients are categorized as follows:

Definite HHT: If they meet three to four of the criteria.

Probable HHT: If they meet two of the criteria.

HHT unlikely: If they meet zero to one of the criteria [3].

Epistaxis associated with HHT is managed using moisturizing topical treatments that add moisture to the nasal lining, thereby reducing nosebleeds. It is recommended to start screening with colonoscopy for individuals with suspected or proven SMAD4-HHT mutation at the age of 15 years. Subsequent screenings every three years in the absence of polyps or every year if colonic polyps are detected are recommended. General population guidelines for colon cancer apply to the remaining HHT (non-SMAD4). Argon plasma coagulation (APC) is the most effective way to treat bleeding lesions and significant nonbleeding lesions ranging from 1 to 3 mm during EGD but repeated sessions should be avoided to prevent repeated damage to the intestinal lining. Intravenous (IV) bevacizumab, vascular endothelial growth factor (VEGF) inhibitor, or other systemic antiangiogenic therapies are applied in individuals experiencing moderate to severe GI bleeding associated with HHT. All individuals with HHT should undergo testing for iron deficiency anemia. If anticoagulation is required, unfractionated heparin, low-molecular-weight heparin, and vitamin K antagonists are preferred over direct-acting oral anticoagulants as they are better tolerated in HHT patients. In individuals with liver AVMs, early referral for liver transplantation should be considered especially if symptomatic complications such as high-output cardiac failure, biliary ischemia, or complicated portal HTN are present [18].

In patients with PAVMs, prognosis depends on treatment which is either peri angiographic embolization techniques or surgery. Surgical resection was the only mode of management for PAVMs in the past which is replaced by pulmonary artery embolization. CAVMs have the highest rate of complications and symptomatic lesions are managed through methods such as microsurgical excision, stereotactic radiotherapy (for lesions smaller than 3 cm in diameter), and embolization [19].

## Conclusions

In summary, HHT is a complex disorder affecting multiple systems and the usual manifestation in the GI tract is AVMs. Management of AVMs depends on the severity of GI bleeding. Interventions such as cutaneous microvascular free flaps and Young's procedure, which targets ALK receptors to regulate VEGF expression been trailed but further research and long-term follow-up are required.
